# Alexithymia in population with depressive disorders and suicidal ideation: results of an observational study

**DOI:** 10.1192/j.eurpsy.2024.744

**Published:** 2024-08-27

**Authors:** P. Martínez, A. R. Campos, T. Jiménez Aparicio, M. Fernández Lozano, M. Merizalde Torres

**Affiliations:** HCUV, Valladolid, Spain

## Abstract

**Introduction:**

In clinical practice, significant delays in requesting help are observed in patients with depressive symptoms and suicidal ideation.

**Objectives:**

The objective of this study was to determine factors associated with the time of untreated illness in a population with depressive disorder attending mental health for the first time in the area of Hospital Clínico Universitario de Valladolid (HCUV).

**Methods:**

Methods: This is an observational study including adult patients of both sexes, referred to their first mental health consultation from their Primary Care Physician, with a picture of depressive symptomatology associated with an identifiable stressor. Informed consent was obtained from the patients and authorized by the Ethics Committee of the HCUV. R Studio ® statistical analysis.

The degree of emotional confusion was quantified with item 1 (“I am often confused about the emotions I feel”) of the Toronto Alexithymia Scale (TAS). This item is scored ( 1-5 )from most severe (1) to least (5). On the other hand, the time in weeks between symptom onset to referral, age and symptom severity according to the Montgomery Scale (MADRS) were recorded.

**Results:**

Results: We present data collected in an initial sample of 278 treated patients, with a female predominance (68%), a MADRS severity score (18.05 ± 5.01) and a calculated time without treatment of 59.66 ± 62.26 weeks (Tables 1,2,3).

A subsample of 72 patients with death ideation was studied, with a female predominance (75%) compared to the overall sample (X2 =1.99, p = 0.1585) (Table 4).

It was also observed that death ideation was higher in younger patients (t = 3.18, p = 0.001907) and with a severe MADRS depression score (t = -7.92, p < 0.0001), however they took a similar length of time to receive mental health treatment (T student t = -1.6605, p = 0.099); (Table 5).

There is no previous published evidence that considers the timing of untreated symptoms. According to test statistics, there are differences in untreated symptom time considering gender and TAS score (Table 6).

**Conclusions:**

Death ideation is a current health problem that deserves attention. In multivariate analysis models, an association with clinical and demographic factors has been found; however, there is up to 20% of the variation in prevalence that is not explained by the aforementioned factors. The factors that determine the time delay in seeking help (treatment delays) have not been studied so far.

In this study we observe how a single variable doesn’t explain the delay in the first visit. The interaction between age, gender, alexithymia and hypoprosexia explains the delay in seeking help, although symptom severity doesn’t seem to be related. These data suggest that unexplained causality in multivariate studies may be related to the interaction between clinical and neuropsychological factors.

**Disclosure of Interest:**

None Declared

